# GPMiner: an integrated system for mining combinatorial *cis*-regulatory elements in mammalian gene group

**DOI:** 10.1186/1471-2164-13-S1-S3

**Published:** 2012-01-17

**Authors:** Tzong-Yi Lee, Wen-Chi Chang, Justin Bo-Kai Hsu, Tzu-Hao Chang, Dray-Ming Shien

**Affiliations:** 1 Department of Computer Science and Engineering, Yuan Ze University, Taoyuan 320, Taiwan; 2 Institute of Tropical Plant Sciences, National Cheng Kung University, Tainan 701, Taiwan; 3 Institute of Bioinformatics and Systems Biology, National Chiao Tung University, Hsin-Chu 300, Taiwan; 4 Department of Multimedia and Game Science, Asia-Pacific Institute of Creativity, Miao-Li 351, Taiwan

## Abstract

**Background:**

Sequence features in promoter regions are involved in regulating gene transcription initiation. Although numerous computational methods have been developed for predicting transcriptional start sites (TSSs) or transcription factor (TF) binding sites (TFBSs), they lack annotations for do not consider some important regulatory features such as CpG islands, tandem repeats, the TATA box, CCAAT box, GC box, over-represented oligonucleotides, DNA stability, and GC content. Additionally, the combinatorial interaction of TFs regulates the gene group that is associated with same expression pattern. To investigate gene transcriptional regulation, an integrated system that annotates regulatory features in a promoter sequence and detects co-regulation of TFs in a group of genes is needed.

**Results:**

This work identifies TSSs and regulatory features in a promoter sequence, and recognizes co-occurrence of *cis*-regulatory elements in co-expressed genes using a novel system. Three well-known TSS prediction tools are incorporated with orthologous conserved features, such as CpG islands, nucleotide composition, over-represented hexamer nucleotides, and DNA stability, to construct the novel Gene Promoter Miner (GPMiner) using a support vector machine (SVM). According to five-fold cross-validation results, the predictive sensitivity and specificity are both roughly 80%. The proposed system allows users to input a group of gene names/symbols, enabling the co-occurrence of TFBSs to be determined. Additionally, an input sequence can also be analyzed for homogeneity of experimental mammalian promoter sequences, and conserved regulatory features between homologous promoters can be observed through cross-species analysis. After identifying promoter regions, regulatory features are visualized graphically to facilitate gene promoter observations.

**Conclusions:**

The GPMiner, which has a user-friendly input/output interface, has numerous benefits in analyzing human and mouse promoters. The proposed system is freely available at http://GPMiner.mbc.nctu.edu.tw/.

## Background

Gene transcription is regulated by transcription factors (TFs) that bind specifically to promoter regions; which is the crucial control region for transcriptional activation of all genes [1]. A typical promoter sequence, which is located near the transcriptional start site (TSS), is believed to comprise short DNA sequences known as regulatory elements, including TF binding sites (TFBSs) [2]. With the vast amount of available genomic data, an increasing need exists for techniques that can rapidly and accurately evaluate sequences for the presence of promoters [3]. Furthermore, some important regulatory motifs, such as the TATA box, CCAAT box, GC box, and INR box, must be annotated in promoter sequences. Further, the presence of CpG islands close to a TSS, statistical properties of proximal and core promoters rather than other genomic sequences, orthologous gene promoters, and restricting a promoter region from using information from mRNA transcripts must be considered [4]. Additionally, some co-regulatory networks describe the set of all significant associations among TFs in regulating common target genes [5]. Accordingly, the combinatorial interaction of TFs is critical in gene regulation.

PlantPAN, a database-assisted system for recognizing co-occurrence of *cis*-regulatory elements in plant co-expressed genes [6], is effective for plant promoter investigations. However, no similar resource exists for identifying co-occurrence TFBSs in a group of mammalian promoters. Veerla *et al*. recently developed SMART software for identifying co-occurring TFBSs in gene set promoters [7]. Nevertheless, this software does not have a user-friendly interface for identifying TSSs with regulatory elements and efficiently analyzing combinatorial TFBSs of a group of promoters. COXPRESdb provides coexpressed gene networks and coexpressed gene lists ordered based on the strength of coexpression for humans and mice [8]. However, COXPRESdb does not analyze TFBSs in co-expressed gene promoters. Although TOUCAN is a Java application for identifying significant *cis*-regulatory elements from sets of co-expressed genes, TOUCAN ignores combinatorial TFBSs analysis [9]. This work develops a novel system, Gene Promoter Miner (GPMiner), for identifying co-occurring TFBSs in a group of gene promoters.

However, the promoter region must be precisely identified before identification of TFBSs co-occurrence. Many databases are useful in collecting numerous TSSs and have promoter prediction tools. The DBTSS is a TSS database established by gathering experimentally identified promoter regions via the oligo-capping method [10]. The Eukaryotic Promoter Database (EPD) is an annotated non-redundant collection of eukaryotic POL II promoters, for which the TSS has been determined experimentally [11]. Various promoter prediction methods have been developed for analyzing gene promoter regions (Table S1, additional file 1). The CpGProD program identifies CpG islands in mammalian promoter regions [12]. The DragonGSF program predicts gene promoters based on information of CpG islands, TSSs and downstream signals of predicted TSSs [13]. The NNPP2.2 program applies a time-delay neural network for promoter annotation of the Drosophila melanogaster genome [14]. The Eponine detects the transcriptional initiation site near the TATA box, together with flanking regions of GC enrichment [15]. To identify TSSs, McPromoter, a statistical method, identifies the eukaryotic polymerase II TSS in genomic DNA [16-18]. The FirstEF uses a set of discriminant functions that can recognize both boundaries of the first exon [19]. The PromoSer method computationally identifies TSSs by considering the alignments of numerous partial and full-length mRNA sequences to those of genomic DNA [20]. The PromH scheme identifies promoters based on conservation of regulatory features in pairs of human/mouse orthologous genes. Another regulatory feature of promoter regions, DNA stability, was investigated for analyzing prokaryotic promoters [21]. Notably, DNA stability is a structural property of the DNA duplex fragment. The minimum free energy of the DNA duplex is calculated based on hydrogen bonding of A-T and C-G pairs. Kanhere *et al*. demonstrated that DNA stability of promoter regions provides a much better clue than other features when determining the location of the TSS [21].

Although numerous computational methods have been developed for identifying promoters of genes in genomic sequences, their outcomes are not satisfactory, especially for promoters lacking a TATA box and CpG islands [1]. Furthermore, many methods have poor predictive specificity, generating many false-positive predictions, or have poor sensitivity. Therefore, this work develops an integrated system, GPMiner, that identifies promoter regions with high predictive sensitivity and specificity. Moreover, GPMiner comprehensively annotates regulatory elements, including TFBSs, CpG islands, tandem repeats, the presence of a TATA box, CCAAT box, or GC box, statistically over-represented sequence patterns, GC content (GC%), and DNA stability. Additionally, GPMiner accurately identifies combinatorial TFBSs in a group of gene promoters.

## Construction and content

**Figure 1:**
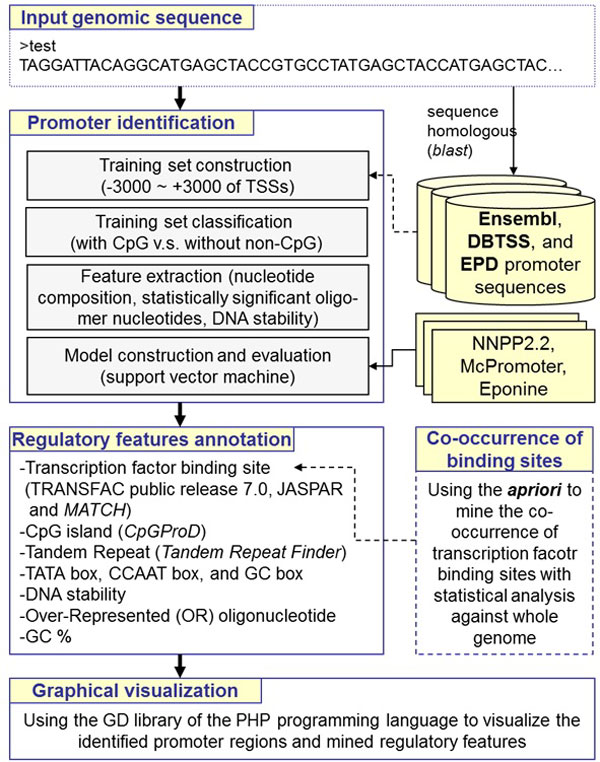
**System flow of GPMiner.**

Figure 1 presents the GPMiner system flow, which identifies promoter regions and annotates transcriptional regulatory features in a user-input genomic sequence. Computational models for promoter identification were constructed by incorporating the support vector machine (SVM) with nucleotide composition features, over-represented hexamer nucleotides, and DNA stability. Additionally, GPMiner allows users to input a group of genes for identification of co-occurring TFBSs in promoter sequences. All mined promoter regions and regulatory features in the user-input sequence are visualized graphically to facilitate analysis of gene transcriptional regulation. The details of the proposed method are as follows.

### Input genomic sequence

Users first input a genomic sequence in the FASTA format to identify promoter regions and to mine regulatory elements within the input sequence. The input sequence is used to search for homogeneity of experimental mammalian promoter sequences collected from the DBTSS (version 6.0) [10], EPD (release 80) [11] and Ensembl (version 61) [22]. All experimentally verified TSSs are using genomic positional information provided by DBTSS and EPD. By default, all the base pairs (bps) starting with the upstream 2000 bps to the downstream 200 bps relative to the TSS (+1) are defined as promoter regions and extracted for a sequence homology search. Notably, GPMiner collects 22774, 25420, 22159, 22475, and 18201 known genes from five mammalian genomes, including the human, mouse, rat, chimpanzee, and dog genomes, respectively. After the sequence homology search, the proposed system outputs a set of known genes with promoter sequences resembling the input sequence. Additionally, users can input the chromosomal location to specify sequence regions for mining regulatory features.

### Promoter identification

**Figure 2:**
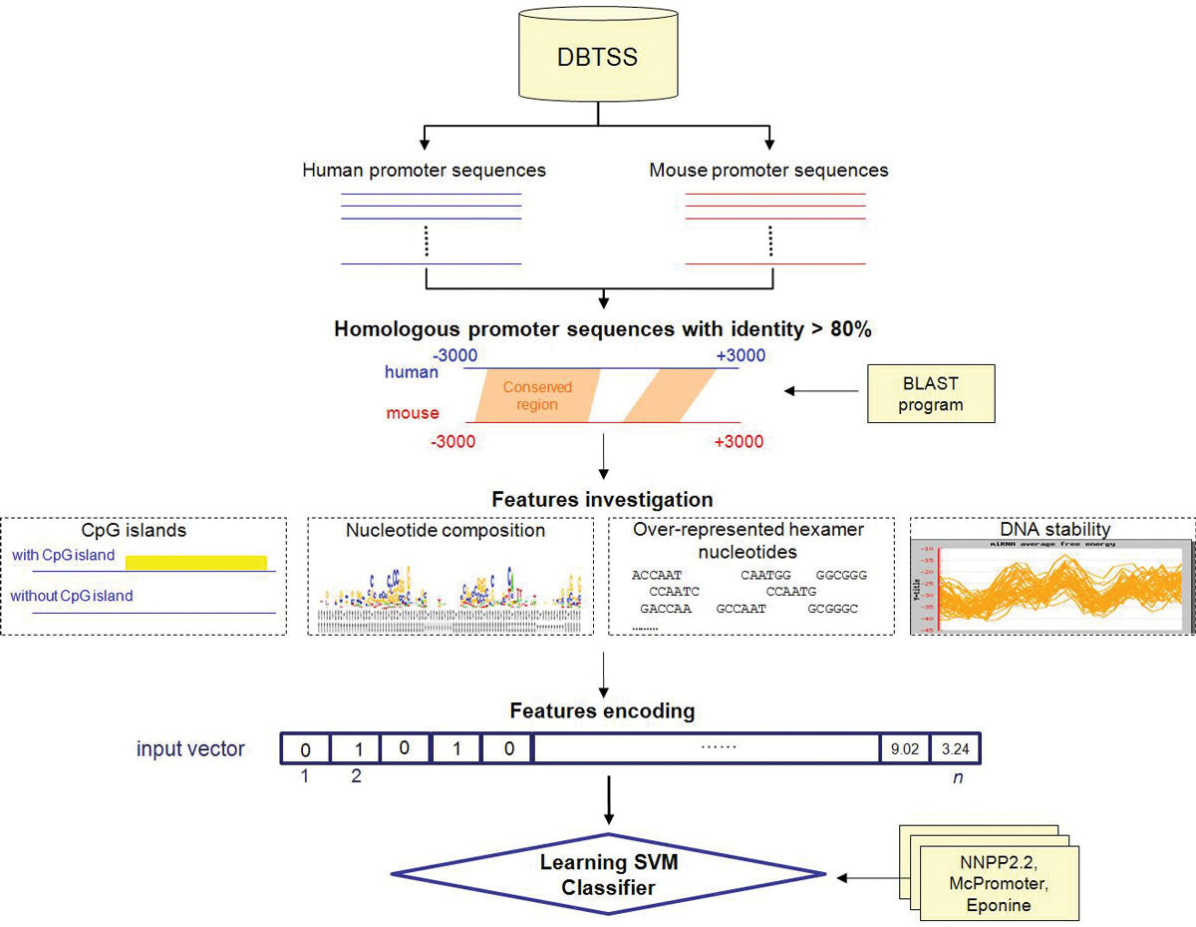
**Analytical flowchart of promoter identification.**

The GPMiner system uses a SVM that considers orthologously conserved regulatory features, such as CpG islands, nucleotide composition, over-represented hexamer nucleotides, and DNA stability, of a promoter sequence to identify mammalian proximal promoters (Figure 2). The promoter length of mammalian cell is usually around 1000 bp [23]. Because some regulatory elements locate far from TSS, numerous *cis*-regulatory elements annotation system used 3000 bp upstream as the maximum region for analysis [24]. Furthermore, several studies indicate the downstream region of TSS play critical roles during transcription. Therefore, 3000 bp downstream of TSS are also selected to analyze. Consequently, experimentally identified promoters originating from human and mouse genomes collected from the DBTSS (Table S2, additional file 1) were mapped to Ensembl genomic positions, and flanking sequences of -3000 bps to +3000 bps around the mapped TSSs were selected. Furthermore, homologous promoter sequences between human and mouse genomes were analyzed using the BLAST program [25]. The sequence identity of homologous promoter sequences exceeding 80% were extracted and defined as training sequences. These training sequences were classified into two subgroups based on whether CpG islands were present by CpGProD [12]. Table S3 (in additional file 1) lists the statistics of the classified training set.

After constructing and classifying the training set, training sequences are first analyzed with their nucleotide composition to calculate the occurrence rate of mono-, di-, and tri-mer nucleotides within a 20-bp window sliding along training sequences. Figure S1 (in additional file 1) lists average distributions of occurrence rates of nucleotide compositions. Pearson's correlation coefficient is calculated for clustering average distributions of mono-, di-, and tri-mer nucleotides into two groups based on the two major distributions of adenine and guanine (Table S4, additional file 1). Furthermore, training sequences are also used to extract over-represented 6-mer nucleotides within a specified window size around the TSSs, which comprise the so-called positive set. The occurrence probabilities of 6-mer nucleotides in the specified window are calculated and compared to background probabilities of the entire genome. By optimizing the number (50-200) of over-represented 6-mer nucleotides, the top 100 over-represented 6-mer nucleotides are selected as training features.

Furthermore, DNA stability is a feature used for identifying promoter sequences. SantaLucia *et al*. [26] used the unified standard free energy of ten dinucleotide duplexes--AA/TT, AT/TA, TA/AT, CA/GT, GT/CA, CT/GA, GA/CT, CG/GC, GC/CG, and GG/CC [26] (Table S5, additional file 1)--to calculate the standard free energy change of a DNA oligonucleotide based on dinucleotide composition. This work applied the equation of standard free energy change to determine the stability of a DNA duplex with a window size of 15 nt sliding from -3000 to +3000, corresponding to the TSSs in training sequences. Figure S2 (in additional file 1) shows distributions of average free energy of DNA duplex formation. Near the TSS, a peak exists in the region starting from -10 to -30, which corresponds to the TATA box in eukaryotic promoter sequences.

A public SVM library LIBSVM [27] is used to construct predictive models. The SVM kernel function is set to the radial basis function (RBF). Before using extracted regulatory features to train SVM models, the specified window sizes of proximal promoter regions, which comprise the so-called positive set, must be defined. Therefore, five window sizes--60 to +20, -100 to +50, -200 to +100, -300 to +150, and -400 to +200--are defined, and a benchmark is applied to evaluate the predictive performance of proximal promoter regions. The benchmark, namely, cross-validation, extracts equal sizes from the positive set and negative set, constructs the SVM model, and evaluates the model with *k*-fold cross-validation. Training sequences within the specified window are defined as the positive set; regions other than those in specified windows, with window sizes equal to those in the positive set, are chosen randomly as the negative set.

Predictive performance of the constructed models is evaluated by five-fold cross-validation [28]. Training data are divided into five groups by splitting each dataset into five approximately equally sized subgroups. During cross-validation, each subgroup is used as the validation set in turn, and the remaining comprise the training set. Next, the measures of predictive performance of trained models are Precision (Prec) = TP/(TP+FP), Sensitivity (Sn) = TP/(TP+FN), Specificity (Sp) = TN/(TN+FP), and Accuracy (Acc) = (TP + TN)/(TP+FP+TN+FN), where TP, TN, FP, and FN are the true positive, true negative, false positive, and false negative predictions, respectively. The constructed SVM models of three different regulatory features are measured, and models with the best predictive accuracy are selected for the mammalian proximal promoter prediction. Moreover, several promoter prediction tools, NNPP2.2 [14], Eponine [15] and McPromoter [16], are integrated into GPMiner to provide additional information about the proximal promoter, thereby improving predictive specificity.

### Mining *cis*-regulatory features

After identifying proximal promoter regions, regulatory elements involving gene transcriptional regulation, such as transcription factor binding sites, CpG islands, the TATA box, CCAAT box, GC box, and over-represented sequences, are annotated. Furthermore, tandem repeats and DNA stability and GC content in the promoter region are provided for advanced analysis of gene transcriptional regulation. Table 1 shows the integrated databases and GPMiner tools for mining regulatory elements within input sequences. For instance, MATCH [29] was utilized for scanning TFBSs in an input sequence using the TF binding profiles from TRANSFAC public release version 7.0 [30] and JASPAR [31]. The CpGProD program [12] was applied to detect the CpG island in a promoter region with a prediction specificity of roughly 70%. A tandem repeat finder [32] was applied to identify tandem repeats in promoter sequences. In detecting the TFBS in promoter regions, cutoff values of core and matrix scores of the MATCH program are set to 1.0 and 0.7, respectively. Particularly, frequent regulatory elements, such as the TATA box, CCAAT box, and GC box, are represented separately.

**Table 1:** Supported regulatory features in GPMiner

Regulatory features	Integrated database or tools	Descriptions
Transcriptional start site	NNPP2.2 [14]	Applying a time-delay neural network for promoter annotation
	
	McPromoter [16]	Using a statistical method to identify eukaryotic polymerase II TSS in genomic DNA
	
	Eponine [15]	Predicting the transcription start site for a DNA sequence with prediction specificity > 70%

Transcription factor (TF) binding site	TRANSFAC public release 7.0 [46]	Storing the experimentally verified transcription factors, their genomic binding sites and DNA-binding profiles
	
	MATCH [29]	Scanning the transcription factor binding site using the transcription factor binding profiles from TRANSFAC public release 7.0 and JASPAR

CpG island	CpGProD [12]	Detecting the CpG island

Repeats	TRF [32]	A tandem repeat finder

TATA box, CCAAT box, and GC box	MATCH [29]	Scanning the TATA-, CCAAT- and GC-box by the transcription factor binding profiles from TRANSFAC
	
	Narang *et al*. [47]	Defining the 6-mer pattern of the TATA box, CCAAT box, and GX box with positional density

Over-represented pattern	Huang *et al*. [48]	Defining the statistically significant pattern in the promoter region

DNA stability	Aditi Kanhere *et al*. [21]	Predicting the DNA stability of the promoter region

Co-occurrence of TF binding sites	apriori [35]	A method to mine the association rules

Conserved regions between homologous gene promoter sequences	Blast [25]	Using the *blast *program to analyze the conserved region between the homologous gene promoter sequences

Several important regulatory features, such as repeats and over-represented oligonucleotides, are integrated. Repeats, such as tandem repeats, *Alu*, and L1 elements can alter OR the methylation distribution in a genome, and possibly in gene transcription [33,34]. The proposed system applies a statistical method to identify over-represented oligonucleotides (6-12 bps) in promoter regions; these over-represented oligonucleotides are identified by comparing their occurrence frequencies in promoter regions with their background occurrence frequencies throughout the whole genome (See additional file 1 for a detailed description). Based on statistical significance, this work chose the oligonucleotide with a *Z*-Score > 5 as the OR sequence. Moreover, DNA stability distributions are provided. The GC contents are also calculated using a window size of 15 nt and used as references for identification of CpG islands.

### Identifying co-occurrence of TFBSs in a group of gene promoters

The GPMiner functionalities allow users to input a group of genes to mine co-occurrence of TFBSs in promoter regions. A mining association rules method, namely, *a priori *[35], is applied to mine the co-occurrence of TFBSs in a group of gene promoter sequences. Consider a large database with transactions, in which each transaction consists of a set of items. An association rule is an expression, such as *A *≥ *B*, where *A *and *B *are item sets. The related mining association rule states that a transaction in a database containing *A *also contains *B*. For example, 90% of people who purchase beer also purchase diapers. Herein, 90% is rule confidence. Support of the *A *≥ *B *rule used is the percentage of transactions containing both *A *and *B*.

The formal problem statement is as follows. Let *S *= {*s*1, *s*2, ..., *sm*} be a set of known TFBSs of the human genome. The union of members in the set *S *is called the item set. Let *G *= {*g*1, *g*2, ..., *gm*} be a group of genes with differential expression in a specific tissue. Each promoter region of a gene is mapped to a transaction containing a set of known regulatory sites, also called items. We assume promoter region *S *contains *A*, a set of items of *I*, when *A *⊆ *S*. An *association rule *is an implication of the relationship *A *≥ *B*, where *A *⊂ *I*, *B *⊂ *I*, and *A *∩ *B *= ϕ. The *A *≥ *B *rule holds in the set of promoter regions *D *with *confidence conf *when *c*% of transactions in *D *contains both *A *and *B*. The *A *≥ *B *rule has *support sup *in the repetitive sequence set *D *when *s*% of promoter regions in *D *contains *A *∪ *B*. The association rules, the so-called co-occurrence of TFBSs, are generated when a rule has higher support and confidence than those specified by a user.

After mining co-occurrences (combinations) of TFBSs in a group of gene promoter sequences, the statistical significance each combination must be examined against the background set of genes using the hypergeometric model:

$$P\left(t\right)=\overset{T}{\underset{t}{\sum }}\frac{C_{t}^{T}\times C_{k-t}^{K-T}}{C_{k}^{K}}$$

where *K *is the number of background gene promoters used, *T *is the number of observed gene promoters input by users, *k *is the number of promoters that have the combination in the background gene set, and *t *is the number of promoters that have the combination in the observed gene set. The *P*-value is calculated for each combination based on the hypermetric equation--the *P*-value decreases, statistical significance increases.

### Graphical visualization

After mining proximal promoter regions and regulatory features, all mined regulatory features are presented graphically in the web interface, which is constructed using the GD library and PHP programming language. To simplify graphical visualization, regulatory features with numerous entries are presented initially in an overview form. Regulatory features are displayed in detail when users click the "detailed view" button. Additionally, detailed information of regulatory features is listed in tabular form. The co-occurrences of TFBSs in a set of gene promoter sequences are also represented graphically. When users investigate promoters of known genes, conserved regions of homologous gene promoters are displayed graphically, as are regulatory features found in conserved promoter regions. The graphical visualization of regulatory elements facilitates analysis of gene transcription regulation.

### Utilities and discussion

#### Performance of promoter identification

A benchmark, namely, cross-validation, is used to evaluate the predictive performance of GPMiner, which incorporates an SVM with nucleotide composition, over-represented hexamer nucleotides, and DNA stability for mammalian proximal promoter identification. The benchmark is used to extract equal sizes of the positive set and negative set, construct the SVM model, and evaluate the model with k-fold cross-validation (k = 5). Table S6 (in additional file 1) lists the prediction performance of the constructed SVM models trained with three different regulatory features based on the five window sizes. Since training sequences are classified into two subgroups by CpG islands--with CpG islands and without CpG islands--predictive performance of group with CpG islands is markedly higher than that of the group without CpG islands; furthermore, as window size increases, the prediction performance of SVM models increases. However, after considering both prediction performance and window size, a window size of -200 to +100 is selected as the specified window for identifying proximal promoter regions. Vertebrate gene expression is frequently regulated by the proximal promoter, which is traditionally defined as between -200 bp and the TSS [36].

Table 2 lists the predictive performance of SVM models trained with combinations of the three different regulatory features, such as over-represented hexamer nucleotides (OR), nucleotide composition (NC), and DNA stability (DS). Three training sets, "all", with CpG islands, and without CpG islands, are evaluated by benchmark cross-validation, and based on the specified window size of 200 to 100 relative to the TSS (+ 1). In all three training sets, the combination OR+NC+DS performs better than other combinations. Moreover, the training set, namely, that with CpG islands, which achieves a predictive accuracy of 82%, performs better than training sets of "all" and without CpG islands. Both SVM models trained with the training sets with CpG islands and without CpG islands are used for proximal promoter identification. Whether an input sequence contains a CpG island is then detected, and the sequence is then predicted by the SVM model with CpG islands or the SVM model without CpG islands.

**Table 2:** The prediction performance of SVM models with combinations of three kinds of regulatory features such as over-represented hexamer nucleotides (OR), nucleotide composition (NC), and DNA stability (DS), is evaluated by benchmark "Cross-validation" based on the specified window size -200 to +100 of TSS(+1).

Training set	Window size	Features	Precision	Sensitivity	Specificity	Accuracy
All (6,452)	-200 ~+100	OR+NC	77%	71%	79%	75%
	-200 ~+100	OR+DS	76%	69%	78%	74%
	-200 ~+100	NC+DS	75%	74%	76%	75%
	-200 ~+100	OR+NC+DS	79%	76%	79%	78%

With CpG (4,898)	-200 ~+100	OR+NC	79%	81%	79%	80%
	-200 ~+100	OR+DS	77%	80%	76%	78%
	-200 ~+100	NC+DS	77%	82%	75%	78%
	-200 ~+100	OR+NC+DS	80%	84%	79%	82%

Without CpG (1,554)	-200 ~+100	OR+NC	68%	70%	67%	68%
	-200 ~+100	OR+DS	68%	71%	66%	68%
	-200 ~+100	NC+DS	66%	67%	66%	66%
	-200 ~+100	OR+NC+DS	69%	69%	71%	70%

The number of training sequences used to construct the SVM models is shown in parenthesis of the column "Training set".

Notably, GPMiner lets users input a novel sequence to annotate the proximal promoter region with the putative TSS. Thus, 1871 human promoter sequences (from -3000 to +3000) in the EPD comprise the independent test set used to evaluate predictive performance. The test sequences whose regions are within -200 to +100 relative to the TSSs (+1) are defined as a positive set; otherwise, the negative set is extracted randomly from regions other than those in the positive set. Table S7 (in additional file 1) compares the predictive performance of GPMiner and those of NNPP2.2, Eponine, and McPromoter. Furthermore, Figure S3 (in additional file 1) shows the distribution of promoter predictions of GPMiner, NNPP2.2, Eponine, and McPromoter. The sensitivity of GPMiner is better than that of the other methods; however, predictive specificity of McPromoter and Eponine are better than that of GPMiner. With consideration of high specificity, NNPP2.2, Eponine, and McPromoter are integrated to reduce the number of false-positive predictions.

#### Web interface

The GPMiner system has two primary functions. First, "gene group analysis" is adopted to identify co-occurrence of TFBSs in a group of gene promoters. Combinatorial regulation by TF complexes is an important feature of eukaryotic gene regulation [5,37,38]. Second, "promoter analysis" can be employed to analyze TFBSs, CpG islands, tandem repeats, the presence of a TATA box, CCAAT box, or GC box, statistically over-represented sequence patterns, GC content (GC%) and DNA stability in the promoter sequence of a given gene ID or a novel promoter sequence. Furthermore, cross-species analysis of homologous gene promoters is performed by GPMiner, such that conserved regulatory features in promoter regions can also be observed.

**Figure 3:**
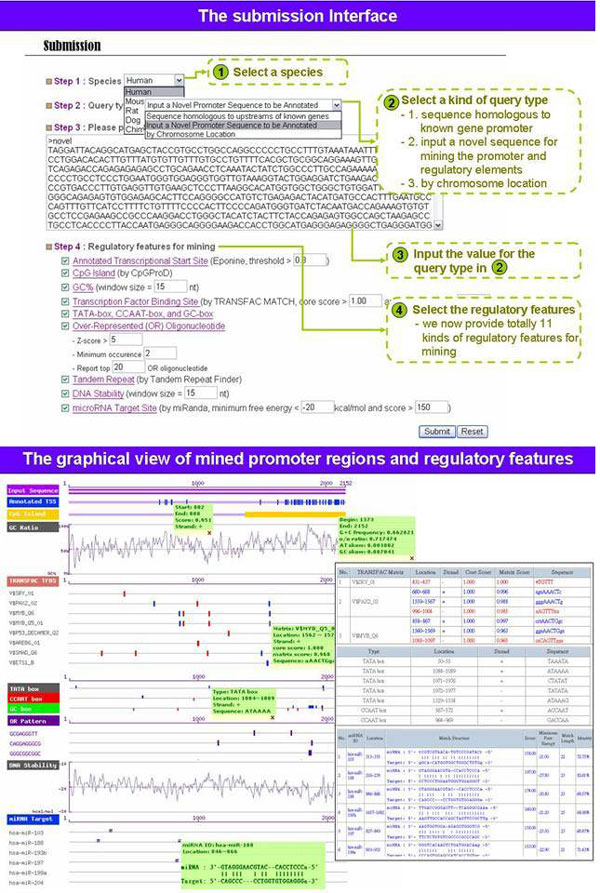
**The submission and result interface of GPMiner.**

Figure 3 shows the web interfaces of GPMiner. In the submission interface, users first choose one of five mammals, such as human, mouse, rat, chimpanzee or dog, and input a genomic sequence or chromosomal location for identifying proximal promoter regions and for mining regulatory features. Eight regulatory features currently exist in GPMiner. By default, all regulatory features are chosen for annotation in the input sequence. Notably, users can input a chromosome location to specify regions of interest for retrieving genes located in this chromosome region. During the mining process, the proposed system uses various tools individually to annotate different regulatory features in an input sequence. Each annotating tool for regulatory features has some search parameters, such as score threshold in NNPP2.2, Eponine, and McPromoter, the core score and matrix score for the MATCH program, *Z*-score for over-represented oligonucleotides, and support and confidence scores for co-occurrence TFBSs analysis, in a gene group search. Default parameters for these tools are set and the related documentation is shown on the help webpage. After mining regulatory features, a graphical visualization of identified promoter regions and mined regulatory features is provided to users. Figures S4 and S5 (see additional file 1) present graphical representations of regulatory elements for known gene promoter and homologous promoter sequences, respectively.

#### Case studies

**Figure 4:**
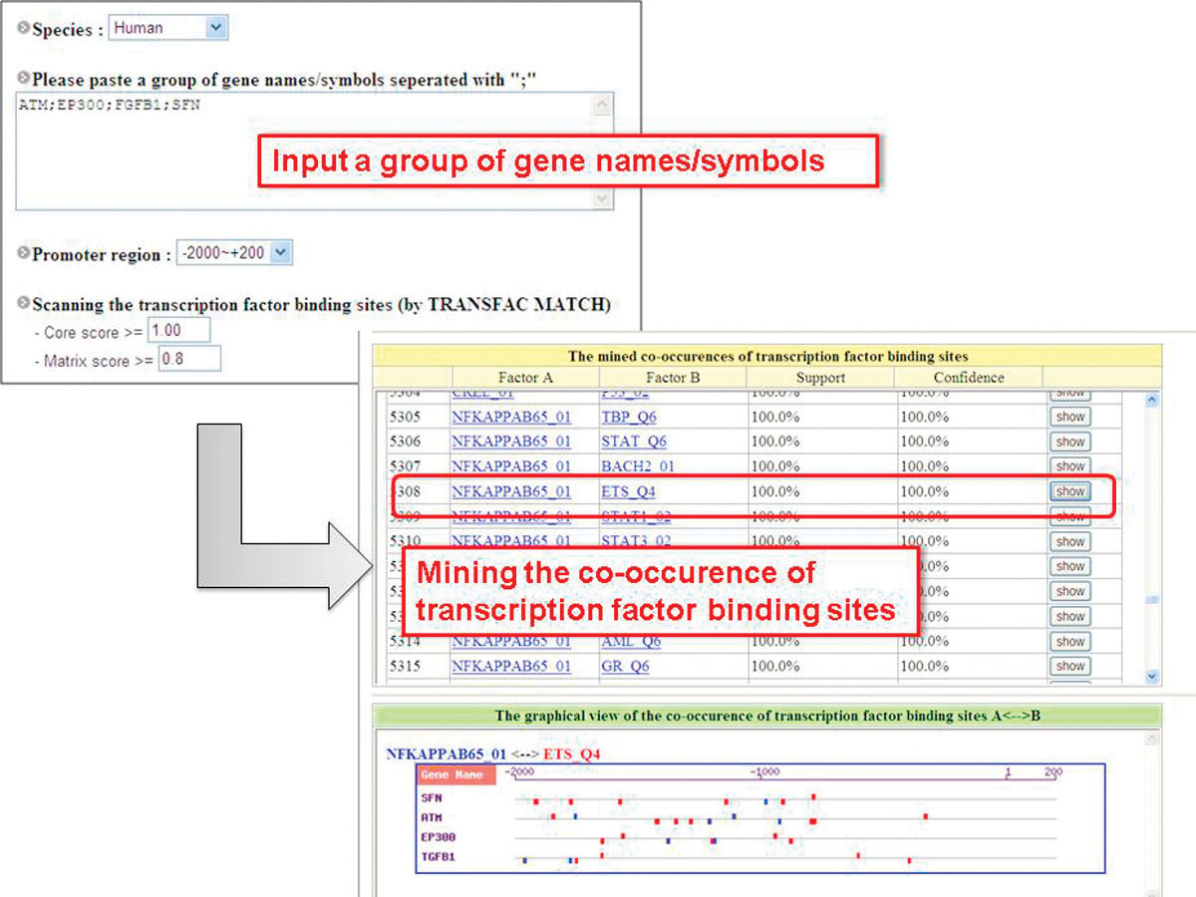
**Gene group analysis in GPMiner.**

Figure 4 shows an example gene group analysis. Notably, NFkappaB is a well-known induced TF that controls kinetically complex patterns of gene expression in multiple pathways in human. In a previous study, ATM, EP300, FGFB1, and SFN were regulated by NF-kappaB and co-regulated by the Ets TF in the progression of various cancers [39]. To effectively apply GPMiner, four gene names were input for gene group analysis by GPMiner to detect co-occurring TFBSs. The thresholds of the core score and matrix score values in TFBS scanning were 1.0 and 0.9, respectively, and the support and confidence values in co-occurrence analysis were set both at 90%. Notably, NF-kappaB and Ets are also identified as combinatorial TFs in these four gene promoters after three analytical steps by GPMiner. This effective result was confirmed by known regulatory pathways [39]. Therefore, GPMiner accurately identifies TFBSs in a set of gene promoters. The proposed system can be applied to analyze co-regulation in microarray gene-expression databases such as COXPRESdb [40] and Genevestigator [41]. The proposed GPMiner system improves our understanding of transcription regulatory networks of gene regulation in mammalians.

Moreover, to demonstrate the application of single promoter analysis, a case study involving humans is described below. The *v-fos FBJ murine osteosarcoma viral oncogene homolog *(gene symbol is FOS) gene is a regulator of cell proliferation, differentiation, and transformation [42]. Through experimentally verified annotation of the Entrez Gene database, the FOS gene is regulated by numerous transcription factors such as SP1, SRF, SAP-1, and AP-1. Additionally, the FOS gene exhibited DNA methylation based on information in the Gene Ontology database. The FOS gene promoter sequence was extracted and input into GPMiner to mine the proximal promoter region and annotate regulatory elements. The DNA stability of the input sequence is graphically represented and the proximal promoter region is highlighted (Figure S2, additional file 1). Using the TSS prediction tool Eponine, potential TSSs are located near positions 500 and 2000 bps. The CpG islands were annotated, as were numerous TFs that may regulate the FOS gene promoter, including SP1, SRF, SAP-1, and AP-1. Moreover, the TATA box was annotated near position 2000 bps. To summarize annotated regulatory features, the proximal promoter region is likely located near 2000 bps since the experimentally validated TSS of the FOS gene was located at 2001 bps.

## Conclusions

The GPMiner system has a gene group analysis function for analyzing the co-occurrence of TFBSs with statistical measures in a set of co-expressed genes. This function uses a practical platform to examine co-expression genes of microarray data in transcriptional regulation networks. Furthermore, the GPMiner system has a user-friendly input/output interface, and has numerous advantages in mammalian promoter analysis. The proposed system incorporates an SVM with nucleotide composition over-represented hexamer nucleotides and DNA stability for mammalian proximal promoter identification and mines regulatory elements, including TSSs, TFBSs, CpG islands, tandem repeats, the TATA box, CCAAT box, GC box, statistically over-represented sequence patterns, GC content (GC%) and DNA stability. Evaluated by benchmark cross-validation, the predictive sensitivity and specificity of GPMiner are roughly 80%. All mined promoter regions and regulatory features in the user input sequence are graphically visualized to facilitate gene transcription analysis. Table 3 compares the functions of several representative programs for promoter annotation with those of GPMiner.

**Table 3:** Comparison of GPMiner with several representative gene promoter annotation programs

Transcriptional regulatory features	**PromoSer **[20]	**PromH **[49]	**DragonGSF **[13]	**McPromoter **[16]	GPMiner
Species supported	Human, mouse, and rat	Human and mouse	Mammalian	Eukaryote	Human, mouse, rat, chimp, and dog
Promoter identification	Yes	Yes	Yes	Yes	Yes
Map to known gene promoters	Yes	-	-	-	DBTSS, EPD and Ensembl
Transcription factor binding site	-	Yes	Yes	-	TRANSFAC public release and JASPAR, MATCH
TATA-box	-	Yes	-	Yes	Yes
Tandem repeat	Yes	-	-	Yes	Tandem Repeat Finder
CpG island	-	-	Yes	-	CpGProD
Over-represented pattern	-	-	-	-	Yes
DNA stability	-	-	-	-	Yes
GC content	-	-	Yes	-	Yes
Co-occurrence of TFBSs	-	Yes	-	-	Yes
Graphical view	Yes	-	-	Yes	Yes

The Functional Annotation of the Mouse 3 (FANTOM3) [43] provides comprehensive experimentally identified TSSs of human and mouse genomes by cap analysis of gene expression (CAGE) [44]. The comprehensive TSSs of CAGE may be used to analyze promoters in advance. In addition to DNA stability, several structural properties of the DNA duplex in the promoter region, such as DNA curvature and bendability [45], should be analyzed and applied to predict identify gene promoter regions in mammals. Future versions of GPMiner will include detailed information about gene regulation such as microarray gene-expression profiles. The GPMiner system will be maintained and updated continuously.

## Availability

The GPMiner web server will be continuously maintained and updated. The web server is now freely available at http://GPMiner.mbc.nctu.edu.tw/.

## Competing interests

The authors declare that they have no competing interests.

## Authors' contributions

TYL and WCC conceived and supervised the project. TYL, JBKH, and DMS was responsible for the design, computational analyses, implemented the web-based tool, and drafted the manuscript with revisions provided by WCC and THC. All authors read and approved the final manuscript.

## Supplementary Material

Additional file 1 **Additional figures and tables.** Contains additional figures and tables showing further results in the study. Click here for file

## References

[B1] Xuan Z Zhao F Wang J Chen G Zhang MQ Genome-wide promoter extraction and analysis in human, mouse, and rat Genome Biol 2005 6 8 R72 10.1186/gb-2005-6-8-r72 16086854 PMC1273639

[B2] Prakash A Tompa M Discovery of regulatory elements in vertebrates through comparative genomics Nat Biotechnol 2005 23 10 1249 1256 10.1038/nbt1140 16211068

[B3] Burden S Lin YX Zhang R Improving promoter prediction for the NNPP2.2 algorithm: a case study using Escherichia coli DNA sequences Bioinformatics 2005 21 5 601 607 10.1093/bioinformatics/bti047 15454410

[B4] Bajic VB Tan SL Suzuki Y Sugano S Promoter prediction analysis on the whole human genome Nat Biotechnol 2004 22 11 1467 1473 10.1038/nbt1032 15529174

[B5] Kato M Hata N Banerjee N Futcher B Zhang MQ Identifying combinatorial regulation of transcription factors and binding motifs Genome Biol 2004 5 8 R56 10.1186/gb-2004-5-8-r56 15287978 PMC507881

[B6] Chang WC Lee TY Huang HD Huang HY Pan RL PlantPAN: Plant promoter analysis navigator, for identifying combinatorial cis-regulatory elements with distance constraint in plant gene groups BMC Genomics 2008 9 561 10.1186/1471-2164-9-561 19036138 PMC2633311

[B7] Veerla S Ringner M Hoglund M Genome-wide transcription factor binding site/promoter databases for the analysis of gene sets and co-occurrence of transcription factor binding motifs BMC Genomics 2010 11 145 10.1186/1471-2164-11-145 20193056 PMC2841680

[B8] Obayashi T Hayashi S Shibaoka M Saeki M Ohta H Kinoshita K COXPRESdb: a database of coexpressed gene networks in mammals Nucleic Acids Res 2008 36 Database issue D77 D82 17932064 10.1093/nar/gkm840 PMC2238883

[B9] Aerts S Thijs G Coessens B Staes M Moreau Y De Moor B Toucan: deciphering the cis-regulatory logic of coregulated genes Nucleic Acids Res 2003 31 6 1753 1764 10.1093/nar/gkg268 12626717 PMC152870

[B10] Yamashita R Suzuki Y Wakaguri H Tsuritani K Nakai K Sugano S DBTSS: DataBase of Human Transcription Start Sites, progress report 2006 Nucleic Acids Res 2006 34 Database issue D86 D89 16381981 10.1093/nar/gkj129 PMC1347491

[B11] Zampieron A Elseviers M De Vos JY Favaretto A Geatti S Harrington M The European practice database (EPD): results of the study in the North-East of Italy EDTNA ERCA J 2005 31 1 49 54 16083029 10.1111/j.1755-6686.2005.tb00391.x

[B12] Ponger L Mouchiroud D CpGProD: identifying CpG islands associated with transcription start sites in large genomic mammalian sequences Bioinformatics 2002 18 4 631 633 10.1093/bioinformatics/18.4.631 12016061

[B13] Bajic VB Seah SH Dragon gene start finder: an advanced system for finding approximate locations of the start of gene transcriptional units Genome Res 2003 13 8 1923 1929 12869582 10.1101/gr.869803 PMC403784

[B14] Reese MG Application of a time-delay neural network to promoter annotation in the Drosophila melanogaster genome Comput Chem 2001 26 1 51 56 10.1016/S0097-8485(01)00099-7 11765852

[B15] Down TA Hubbard TJ Computational detection and location of transcription start sites in mammalian genomic DNA Genome Res 2002 12 3 458 461 10.1101/gr.216102 11875034 PMC155284

[B16] Ohler U Promoter prediction on a genomic scale--the Adh experience Genome Res 2000 10 4 539 542 10.1101/gr.10.4.539 10779494 PMC310866

[B17] Ohler U Liao GC Niemann H Rubin GM Computational analysis of core promoters in the Drosophila genome Genome Biol 2002 3 12 RESEARCH0087 12537576 10.1186/gb-2002-3-12-research0087 PMC151189

[B18] Ohler U Harbeck S Niemann H Noth E Reese MG Interpolated markov chains for eukaryotic promoter recognition Bioinformatics 1999 15 5 362 369 10.1093/bioinformatics/15.5.362 10366656

[B19] Davuluri RV Grosse I Zhang MQ Computational identification of promoters and first exons in the human genome Nat Genet 2001 29 4 412 417 10.1038/ng780 11726928

[B20] Halees AS Leyfer D Weng Z PromoSer: a large-scale mammalian promoter and transcription start site identification service Nucleic Acids Res 2003 31 13 3554 3559 10.1093/nar/gkg549 12824364 PMC168956

[B21] Kanhere A Bansal M A novel method for prokaryotic promoter prediction based on DNA stability BMC Bioinformatics 2005 6 1 1 10.1186/1471-2105-6-1 15631638 PMC545949

[B22] Hubbard T Andrews D Caccamo M Cameron G Chen Y Clamp M Clarke L Coates G Cox T Cunningham F Ensembl 2005 Nucleic Acids Res 2005 33 Database issue D447 D453 15608235 10.1093/nar/gki138 PMC540092

[B23] Vandenbon A Nakai K Modeling tissue-specific structural patterns in human and mouse promoters Nucleic Acids Res 2010 38 1 17 25 10.1093/nar/gkp866 19850720 PMC2800225

[B24] Ji X Li W Song J Wei L Liu XS CEAS: cis-regulatory element annotation system Nucleic Acids Res 2006 34 Web Server issue W551 W554 10.1093/nar/gkl322 16845068 PMC1538818

[B25] Altschul SF Gish W Miller W Myers EW Lipman DJ Basic local alignment search tool J Mol Biol 1990 215 3 403 410 2231712 10.1016/S0022-2836(05)80360-2

[B26] SantaLucia J Jr A unified view of polymer, dumbbell, and oligonucleotide DNA nearest-neighbor thermodynamics Proc Natl Acad Sci U S A 1998 95 4 1460 1465 10.1073/pnas.95.4.1460 9465037 PMC19045

[B27] Chang C-C Lin C-J LIBSVM: a library for support vector machines 2001 Software available at http://www.csie.ntu.edu.tw/~cjlin/libsvm/

[B28] Chou KC Shen HB Recent progress in protein subcellular location prediction Anal Biochem 2007 370 1 1 16 10.1016/j.ab.2007.07.006 17698024

[B29] Kel AE Gossling E Reuter I Cheremushkin E Kel-Margoulis OV Wingender E MATCH: a tool for searching transcription factor binding sites in DNA sequences Nucleic Acids Res 2003 31 13 3576 3579 10.1093/nar/gkg585 12824369 PMC169193

[B30] Wingender E Karas H Knüppel R TRANSFAC database as a bridge between sequence data libraries and biological function Pac Symp Biocomput 1997 477 485 9390316

[B31] Sandelin A Alkema W Engstrom P Wasserman WW Lenhard B JASPAR: an open-access database for eukaryotic transcription factor binding profiles Nucleic Acids Res 2004 32 Database issue D91 D94 14681366 10.1093/nar/gkh012 PMC308747

[B32] Benson G Tandem repeats finder: a program to analyze DNA sequences Nucleic Acids Res 1999 27 2 573 580 10.1093/nar/27.2.573 9862982 PMC148217

[B33] Batzer MA Deininger PL Alu repeats and human genomic diversity Nat Rev Genet 2002 3 5 370 379 10.1038/nrg798 11988762

[B34] Han JS Szak ST Boeke JD Transcriptional disruption by the L1 retrotransposon and implications for mammalian transcriptomes Nature 2004 429 6989 268 274 10.1038/nature02536 15152245

[B35] Srikant R Vu Q Agrawal R Mining generalized association rules Proceedings of 21st International Conference on Very Large Databases 1995 407 419

[B36] FitzGerald PC Shlyakhtenko A Mir AA Vinson C Clustering of DNA sequences in human promoters Genome Res 2004 14 8 1562 1574 10.1101/gr.1953904 15256515 PMC509265

[B37] Balaji S Babu MM Iyer LM Luscombe NM Aravind L Comprehensive analysis of combinatorial regulation using the transcriptional regulatory network of yeast J Mol Biol 2006 360 1 213 227 10.1016/j.jmb.2006.04.029 16762362

[B38] Yu X Lin J Masuda T Esumi N Zack DJ Qian J Genome-wide prediction and characterization of interactions between transcription factors in *Saccharomyces cerevisiae* Nucleic Acids Res 2006 34 3 917 927 10.1093/nar/gkj487 16464824 PMC1361616

[B39] De Siervi A De Luca P Moiola C Gueron G Tongbai R Chandramouli GV Haggerty C Dzekunova I Petersen D Kawasaki E Identification of new Rel/NFkappaB regulatory networks by focused genome location analysis Cell Cycle 2009 8 13 2093 2100 10.4161/cc.8.13.8926 19502793 PMC2809250

[B40] Obayashi T Kinoshita K COXPRESdb: a database to compare gene coexpression in seven model animals Nucleic Acids Res 2011 39 Database issue D1016 1022 21081562 10.1093/nar/gkq1147 PMC3013720

[B41] Zimmermann P Hirsch-Hoffmann M Hennig L Gruissem W GENEVESTIGATOR. *Arabidopsis *microarray database and analysis toolbox Plant Physiol 2004 136 1 2621 2632 10.1104/pp.104.046367 15375207 PMC523327

[B42] Bakin AV Curran T Role of DNA 5-methylcytosine transferase in cell transformation by fos Science 1999 283 5400 387 390 10.1126/science.283.5400.387 9888853

[B43] Bono H Kasukawa T Furuno M Hayashizaki Y Okazaki Y FANTOM DB: database of Functional Annotation of RIKEN Mouse cDNA Clones Nucleic Acids Res 2002 30 1 116 118 10.1093/nar/30.1.116 11752270 PMC99137

[B44] Carninci P Sandelin A Lenhard B Katayama S Shimokawa K Ponjavic J Semple CA Taylor MS Engstrom PG Frith MC Genome-wide analysis of mammalian promoter architecture and evolution Nat Genet 2006 38 6 626 635 10.1038/ng1789 16645617

[B45] Kanhere A Bansal M Structural properties of promoters: similarities and differences between prokaryotes and eukaryotes Nucleic Acids Res 2005 33 10 3165 3175 10.1093/nar/gki627 15939933 PMC1143579

[B46] Wingender E Chen X Hehl R Karas H Liebich I Matys V Meinhardt T Pruss M Reuter I Schacherer F TRANSFAC: an integrated system for gene expression regulation Nucleic Acids Res 2000 28 1 316 319 10.1093/nar/28.1.316 10592259 PMC102445

[B47] Narang V Sung WK Mittal A Computational modeling of oligonucleotide positional densities for human promoter prediction Artif Intell Med 2005 35 1-2 107 119 10.1016/j.artmed.2005.02.005 16076553

[B48] Huang HD Horng JT Sun YM Tsou AP Huang SL Identifying transcriptional regulatory sites in the human genome using an integrated system Nucleic Acids Res 2004 32 6 1948 1956 10.1093/nar/gkh345 15051813 PMC390354

[B49] Solovyev VV Shahmuradov IA PromH: Promoters identification using orthologous genomic sequences Nucleic Acids Res 2003 31 13 3540 3545 10.1093/nar/gkg525 12824362 PMC168932

