# Supplementary material for: GPMiner: an integrated system for mining combinatorial cis-regulatory elements in mammalian gene group
Source: BMC Genomics. 2012 Jan 17;13(Suppl 1):S3. doi: 10.1186/1471-2164-13-S1-S3 (PMC3587379; doi:10.1186/1471-2164-13-S1-S3)
Supplement: Additional file 1 — Additional figures and tables. Contains additional figures and tables showing further results in the study. [file 1471-2164-13-S1-S3-S1.pdf]

SUPPLEMENTARY MATERIALS

The system integrated numerous computational methods to comprehensively annotate the regulatory features of five mammalian genomes such as human, mouse, rat, chimp, and dog. The related information of the regulatory features such as Transcription Start Site (TSS), first exon end position, Transcription Factor Binding Site (TFBS), CpG island, G + C content, repeats (SINE, LINE, tandem repeats and so on), TATA box, CCAAT box, GC box, statistical over-represented oligonucleotide, DNA stability, microRNA target sites and Single Nucleotide Polymorphism (SNP) are described as follows.

Transcription Start Site

The transcription start site (TSS) is an initiation site of the production of mRNA molecules. The important regulatory elements usually located near the TSS, which is the so called gene promoter region. The system collected five mammalian known gene start sites from Ensembl genome database, including human, mouse, rat, chimpanzee and dog, and the number of known genes are 22774, 25420, 22159, 22475 and 18201, respectively. Since users input a sequence to be searched for the homogeneity with the known gene promoter sequences, the Ensembl annotated start sites are used to extract the promoter region. By default, the upstream 2000 bps from TSS (+1) to the first exon end are extracted and defined as the promoter region. Besides, DBTSS collects a full-length cDNA library which experimentally determined human and mouse gene TSS, are integrated by GPMiner to improve the annotation of gene start sites.

Users can also input a novel sequence to be annotated the putative TSS. The system integrates the TSS prediction tool, Eponine, which detects the transcriptional initiation site near the TATA box together with the flanking regions of G-C enrichment. A parameter of score threshold should be set (0 ~ 1.0), the value is set to 0.8 with the highly prediction accuracy. A lower score threshold will make much TSS predictions, and increase the false positive problem. NNPP2.2, which applied a time-delay neural network for promoter annotation, is integrated in this work. McPromoter, which applied a statistical method to identify eukryotic polymerase II TSS in genomic DNA, is integrated to reduce the false positive predictions.

First Exon End Position (only for known genes)

The information of first exon end position is only annotated for the known genes. With the Ensembl core libraries, the most 5'-end exon of known genes are extracted and used to determine the first exon end position. The average distances between TSS (+1) and first exon end of human, mouse, rat, chimpanzee and dog are 308,

369, 300, 310 and 246, respectively. The information of first exon end position is used by the system to define the known gene promoter regions.

CpG Island

In vertebrate genomes, the CpG Islands (CGIs) are involved in DNA methylation of gene transcription. 50-60% of the human genes exhibit a CGI over the transcription start site (TSS) but not all the CGIs are associated with promoter regions (Larsen *et al.*, 1992). The CGIs associated with promoters can be, *a priori*, identified from their structural characteristics (greater size, higher G+C content and CpG o/e ratio; Ioshikhes and Zhang, 2000; Ponger *et al.*, 2001). CpGProD can detect the CGIs in the promoter region with prediction specificity ~ 70%, which is integrated by GPMiner to search the CGIs for input sequence. The CGIs are defined as DNA regions longer than 500 nucleotides, with a moving average C+C frequency above 0.5 and a moving average CpG observed/expected (o/e) ratio greater than 0.6. The information of CpG islands can help improving the prediction of gene promoter regions.

G + C Content

The C + C content represent a frequency of nucleotide G and C occurrence in a given window. The default window size is 15 nt sliding 1 nt each time. The representation of G + C content can help observing the CpG islands and GC box in the promoter region. It is found that most genes had high G + C content in promoter regions.

Transcription Factor Binding Site

The experimentally identified TF bind sites were obtained from TRANSFAC (professional 8.1), which contains 5,711 transcription factors and 14,406 binding sites. In the system, 4,206 known binding sites are matched to upstream regions of human, mouse, rat, chimpanzee, and dog genes. A program, namely MATCH, was implemented to match the consensus patterns of the TRANSFAC known binding sites to the input sequences. Two important parameters of MATCH, core score and matrix score, represent the sequence matching score of core region and whole region of binding site, respectively. For high specificity of transcription factor binding site matching, the system set 1.0 (perfect match) for core score and 0.95 for matrix score. The known TF binding sites are used to scan the input sequence in both strands, and the positions of each known site homolog are then displayed in the graphical visualization.

TATA box, CCAAT box, and GC box

Narang *et al*. used computational method to reveal several important core and proximal promoter elements such as TATA box, CCAAT box, GC box, etc., along with their expected locations around the TSS. These oligonucleotides are kinds of transcription factor binding site and located near the transcription start site. As shown in Table S8, the lists of TATA box, CCAAT box, and GC box with positional densities are used by GPMiner to help the annotation of promoter region.

Over Represented (OR) Oligonucleotide

The system applies a statistical method to discover statistically significant oligonucleotides in promoter region, the so called over-represented (OR) Oligonucleotide, which is identified by comparing their frequencies of occurrence in the promoter regions to their background frequencies of occurrence throughout whole genome. If $P_b(S)$ is the background occurrence probability of oligonucleotide S in whole genomic sequence, then the oligonucleotide S would be expected to occur $u = T \times P_b(S)$ times in the promoter regions of genes, where T represents the total number of possible matching positions of an oligonucleotide with length w across both strands of the sequence set. Using the binomial distribution model, the standard deviation of oligonucleotide occurrences becomes $\sigma = \{T \times P_b(S) \times [1 - P_b(S)]\}^{1/2}$. Let n be the frequency of the considered oligonucleotide S occurring in the promoter regions; the Z-score is given by Z = (n - u) / σ. The probability of observing at least n successes, as given by Chebyshev's theorem, is less than or equal to $p = [(n-u)/\sigma]^{-2}$. If Z > 0, then a lower p-value corresponds to a more over-represented oligonucleotide. If Z < 0, then a lower p-value corresponds to a more under-represented oligonucleotide. By statistical significance, we selected the oligonucleotide with the p-value (cumulative hypergeometric probability) < 0.01 to be the OR aligonucleotides.

Repeats

The repeats such as SINE, LINE, Alu, L1, and so on, are extracted from Ensembl database by using Ensembl core libraries. Previous study (Batzer *et al.*) found that repeats such as *Alu* and L1 elements can alter the distribution of methylation in the genome, and possibly in gene transcription. These repeats are represented only for known gene promoter sequences. To find the tandem repeats in promoter region, the system integrates a program namely tandem repeat finer. The parameters such as period size, copy number, consensus size, score, etc. are set corresponding to the default value of tandem repeat finer.

DNA Stability

Aditi Kanhere *et al.* [1] devised a novel regulatory feature, DNA stability, for prokaryotic promoter prediction. DNA stability is the structural property of the fragment of DNA duplex, and calculates the minimum free energy based on the hydrogen bond of A-T and C-G pairs. The standard free energy change (ΔG_{37}^{0}) corresponding to the melting transition of an 'n' nucleotide (or 'n-1' dinucleotides) long DNA molecule, from double strand to single strand, is calculated as follows [2]:

$$\Delta G^{0}(total) = -(\Delta G_{ini}^{0} + \Delta G_{sym}^{0}) + \sum_{i=1}^{n-1} \Delta G_{i,i+1}^{0}$$

where, ΔG_{ini}^{0} denotes two types of initiation free energy : "initiation with terminal G·C" and "initiation with terminal A·T"; ΔG_{sym}^{0} is +0.43 kcal/mol and is applicable if the duplex is self-complementary, and $\Delta G_{i,j}^{0}$ represents the standard free energy change for type ij dinucleotide. Table S5 lists the standard free energy changes for ten Watson-Crick types ij.

In the present calculation, each promoter sequence is divided into overlapping windows of 15 bp (or 14 dinucleotide steps), and for each window the free energy is calculated as shown above. This study used the equation of standard free energy change (mentioned in the supplementary materials) to calculate the stability of DNA duplex with window size = 15 nt, sliding from -1000 to +201 of TSS in the DBTSS human and mouse experimentally determined promoters. Figure S1 shows the distributions of average free energy of DNA duplex formation, and reveals a peak near the TSS, lying between -10 and -30 region, which corresponds to the TATA box in the eukaryotic promoter sequences. Aditi Kanhere *et al.* [1] demonstrated that the change in DNA stability appears to provide a much better clue than the usual sequence motifs.

Homologous Gene Promoter Analysis

For cross species analysis of homologous gene promoter sequences, the Ensembl core libraries [3] were used to identify homologous genes among human, mouse, rat, chimpanzee, and dog. These homologous genes are analyzed based on gene sequence similarity, and the paired homologous genes with both sequence coverage and sequence identity exceeding 80% are further analyzed in homologous gene promoter sequences. The statistics of pair of homologous genes among five species considered in this work are given in Table S9. Following the determination of the paired homologous gene sequences among those five mammals, the multiple sequences alignment tool, CLUSTALW, was used to analyze the promoter sequences of the paired homologous genes. This work found that certain pairs of promoter sequences were not conserved while their homologous gene sequences

were highly conserved. Based on the conservation of homologous gene promoter sequences among the five mammals, the conserved regulatory features should have a greater influence on gene transcriptional regulation.

Table S1. The summary of previous promoter prediction programs.

Tool	Method	Species	Features	Data Source	Citation	Sn.	Sp.	False positive rate	Acc.	Available
Eponine	Relevance Vector Machine (RVM)	Mammalian	TATA box in a G+C rich domain	EPD	Reese 2001	53.5%	73.5%	-	-	Yes
Promoter 2.0	ANN	Vertebrate	Four TFBSs (TATA box, CCAAT box, GC box, Inr)	EPD	Ohler, Stemmer et al. 2000	68%	-	8%	-	No
NNPP 2.2	ANN	Drosophila	TATA box & Inr	EPD	Down and Hubbard 2002	70%	-	7.2%	-	Yes
CpGProD	Statistics based	mammalian	CpG Island	GenBank	Ponger and Mouchiroud 2002	56%	39%	-	-	Yes
PromoterInspector	Statistics based	Vertebrate	IUPAC	EPD	Scherf, Klingenhoff et al. 2000	48%	85%	-	-	No
Dragon PF	ANN	Human Chr. 22	CpG Island related	EPD	Knudsen 1999	60.17%	-	-	-	No
Dragon GSF	ANN	Human Chr. 4,21,22	G+C rich &G+C poor	DBTSS	Bajic, Seah et al. 2002	65.10%	-	-	77.80%	No
McPromoter	ANN, Interpolated Markov Model	Human Chr. 22	Statistical properties of promoters versus nonpromoters	EPD	Ohler, et al. 2002	52.1%	40.3%	-	-	Yes
First Exon Finder	Quadratic discriminating analysis	Human Chr. 21,22	CpG Island related	NCBI	Davuluri, Grosse et al. 2001	79.3%	53.5%	-	-	No

Table S2. The statistics of experimentally identified TSSs collected from DBTSS and EPD.

Database	Number of TSSs		
	Human	Mouse	Rat
DBTSS	30,964	19,924	N/A
EPD	1,871	196	119

Table S3. The statistics of training set (-3000 ~ +3000 of TSS) collected from DBTSS. The homologous promoter sequences (sequence identity > 80%) between human and mouse are analyzed by CLUSTALW [4].

**Species**	**Number of training set**		
	**all**	**with CpG**	**without CpG**
Human	6,464	4,898	1,566
Mouse	8,885	6,723	2,162
Homology between human and mouse	6,452	4,898	1,554

Table S4. The lists of high correlation coefficient (CC) of average distributions of mono-, di-, and tri-mer nucleotides based on Adenine and Guanine.

Data set	CC with A >0.8	CC with C >0.8	Not selected
All	T,AA,AT,AC,TA,TT,CA,AAA,AAT,AAC,AAG,ATA,ATC,ATT,ATG,ACA,ACT,AGA,TAA,TAC,TAT,TAG,TTA,TTT,TCA,TGA,CAA,CAT,CTA	G,CC,CG,GC,GG,ACG,AGC,TCC,TCG,CTC,CCC,CCG,CGA,CGT,CGC,CGG,GAC,GTC,GCC,GCG,GGA,GGC,GGG	AG,TC,TG,CT,GA,GT,CC, AGT,AGG,TTC,TTG,TCT,TGT,TGC,TGG,CAC,CAG,CTT,CTG,CCA,CCT,GAA,GAT,GAC,GAG,GTT,TG,GCA,GCT,GGT
with CpG	T,AA,AT,AC,TA,TT,CA,AAA,AAT,AAC,AAG,ATA,ATC,ATT,ATG,ACA,ACT,AGA,AGT,TAA,TAC,TAT,TAG,TTA,TTT,TCA,TCT,TGA,CAA,CAT,CTA,GAA,GAT,GTA	G,CC,CG,GC,GG,ACG,AGC,TCC,TCG,CTC,CCC,CCG,CGA,CGT,CGC,CGG,GAC,GTC,GCC,GCG,GGA,GGC,GGG	AG,TC,TG,CT,GA,GT,ACC,AGG,TTC,TTG,TGT,TGC,TGG,CAC,CAG,CTT,CTG,CCA,CCT,GAG,GTT,GTG,GCA,GCT,GGT
without CpG	AA,AT,TA ,AAA,ATA	G,AG,CC,CG,CT,GC,GG,TC,ACG,AGC,AGG,CAG,CCA,CCC,CCG,CCT,CGA,CGT,CGC,CGG,CTC,CTG,GAC,GAG,GCC,GCG,GCT,GGA,GGC,GGG,GTC,TCC,TGC,TGG	T,AC,CA,GA,GT,TG,TT,AAC,AAG,AAT,ACA,ACC,ACT,AGA,AGT,ATC,ATG,ATT,CAA,CAC,CAT,CTA,CTT,GAA,GAT,GCA,GGT,GTA,GTG,GTT,TAA,TAC,TAG,TAT,TCA,TCT,TGA,TGT,TTA,TTC,TTG,TTT

Table S5. Watson-Crick type unified standard free energy change [5].

Dinucleotide Types	Standard free energy (kcal/mol)
AA/TT	-1.00
AT/TA	-0.88
TA/AT	-0.58
CA/GT	-1.45
GT/CA	-1.44
CT/GA	-1.28
GA/CT	-1.30
CG/GC	-2.17
GC/CG	-2.24
GG/CC	-1.84
Initiation with terminal G·C	0.98
Initiation with terminal A·T	1.03

Table S6. The prediction performance of the constructed SVM models with three kinds of regulatory features based on five specified window sizes.

**Nucleotide Composition**					
**Training set**	**Window size**	**Pr.**	**Sn.**	**Sp.**	**Ac.**
all	- 60 ~ + 20	69%	69%	69%	69%
	-100 ~ + 50	70%	67%	71%	70%
	-200 ~ +100	72%	69%	74%	71%
	-300 ~ +150	74%	70%	75%	72%
	-400 ~ +200	74%	72%	75%	74%
With CpG	- 60 ~ + 20	70%	74%	68%	71%
	-100 ~ + 50	71%	75%	69%	72%
	-200 ~ +100	73%	76%	71%	74%
	-300 ~ +150	74%	77%	74%	75%
	-400 ~ +200	76%	80%	75%	77%
without CpG	- 60 ~ + 20	68%	61%	71%	66%
	-100 ~ + 50	66%	62%	68%	65%
	-200 ~ +100	68%	65%	69%	67%
	-300 ~ +150	67%	63%	68%	66%
	-400 ~ +200	66%	63%	68%	66%
**Over-represented hexa-mer oligonucleotides**					
**Training set**	**Window size**	**Pr.**	**Sn.**	**Sp.**	**Ac.**
all	- 60 ~ + 20	69%	51%	77%	64%
	-100 ~ + 50	71%	58%	76%	67%
	-200 ~ +100	73%	62%	77%	70%
	-300 ~ +150	76%	64%	80%	72%
	-400 ~ +200	79%	65%	83%	74%
with CpG	- 60 ~ + 20	70%	56%	75%	66%
	-100 ~ + 50	72%	67%	74%	71%
	-200 ~ +100	76%	75%	76%	76%
	-300 ~ +150	78%	77%	78%	78%
	-400 ~ +200	81%	79%	82%	80%
without CpG	- 60 ~ + 20	59%	22%	85%	53%
	-100 ~ + 50	65%	28%	85%	56%
	-200 ~ +100	66%	32%	83%	58%
	-300 ~ +150	66%	32%	83%	58%
	-400 ~ +200	66%	35%	82%	58%
**DNA stability**					
**Training set**	**Window size**	**Pr.**	**Sn.**	**Sp.**	**Ac.**
all	- 60 ~ + 20	69%	67%	69%	68%
	-100 ~ + 50	70%	68%	71%	69%
	-200 ~ +100	73%	70%	74%	71%
	-300 ~ +150	72%	70%	72%	71%
	-400 ~ +200	73%	71%	74%	72%
with CpG	- 60 ~ + 20	71%	74%	70%	71%
	-100 ~ + 50	72%	75%	71%	73%
	-200 ~ +100	73%	76%	73%	75%
	-300 ~ +150	75%	79%	73%	76%
	-400 ~ +200	75%	81%	74%	77%
without CpG	- 60 ~ + 20	66%	62%	68%	65%
	-100 ~ + 50	66%	62%	69%	65%
	-200 ~ +100	67%	64%	68%	66%
	-300 ~ +150	67%	65%	69%	66%
	-400 ~ +200	68%	66%	70%	67%

Table S7. The prediction performance of GPMiner comparing with NNPP2.2, Eponine, and McPromoter is evaluated by 1,871 human promoter sequences (-3000 to +3000) of EPD. The testing sequences whose regions within -200 to +100 relative to TSSs (+1) are defined as positive set; otherwise, the negative set is randomly extracted from the regions other than positive set.

Methods	Pr.	Sn.	Sp.	Ac.
GPMiner (OR+NC+DS)	72%	92%	64%	78%
NNPP2.2	55%	67%	45%	56%
Mcpromoter 2.0	72%	5%	97%	51%
Eponine	74%	32%	85%	58%

Table S8. The lists of TATA box, CCAAT box, and GC box with positional densities (Narang *et al.*).

Consensus	Preferred Position	Corresponding oligonucleotides	Window Position	Probability
TATA box	-35 to -25	TATAAA	-40 to -20	0.564
		TATAAC	-40 to -20	0.25
		TATAAG	-40 to -20	0.473
		TATATA	-40 to -20	0.365
		TAAAAG	-40 to -20	0.364
		TAAAGG	-40 to -20	0.299
		TAAATA	-40 to -20	0.275
		TGTATA	-40 to -20	0.307
		ATAAAA	-40 to -20	0.299
		ATAAAG	-40 to -20	0.348
		ATAAAT	-40 to -20	0.285
		ATATAA	-40 to -20	0.394
		CCTATA	-40 to -20	0.437
		CTATAA	-40 to -20	0.597
		CTATAT	-40 to -20	0.413
		GCTATA	-40 to -20	0.543
		GTATAA	-40 to -20	0.568
		GTATAT	-40 to -20	0.331
CCAAT box	-165 to -40 (-90 mean)	ACCAAT	-140 to -80	0.259
		CAATGG	-140 to -80	0.201
		CCAATC	-140 to -80	0.201
		CCAATG	-140 to -80	0.279
		GACCAA	-140 to -80	0.209
		GCCAAT	-140 to -80	0.232
GC box	-164 to +1	GGCGGG	-140 to -80	0.203
		GGGCGG	-140 to -80	0.208
		GGGGCG	-140 to -80	0.218
		CGGCGG	-80 to -20	0.201
		CGGGGC	-80 to -20	0.256
		GCGCCG	-80 to -20	0.203
		GCGGCG	-80 to -20	0.201
		GCGGGC	-80 to -20	0.211
		GCGGGG	-80 to -20	0.253
		GGCGGG	-80 to -20	0.275
		GGGGCG	-80 to -20	0.266
		CGGCGG	-20 to +40	0.249
		GCGGCG	-20 to +40	0.251
		GGCGGC	-20 to +40	0.254

Table S9. Statistics of paired homologous known genes among human, mouse, rat, chimpanzee, and dog with the coverage and identity of the gene sequences both exceed 80%.

**Species**	Human	Mouse	Rat	Chimpanzee	Dog
Human		10,047	9,129	14,082	11,006
Mouse			1,4510	6,883	8,842
Rat				6,365	8,048
Chimpanzee					7,384
Dog					

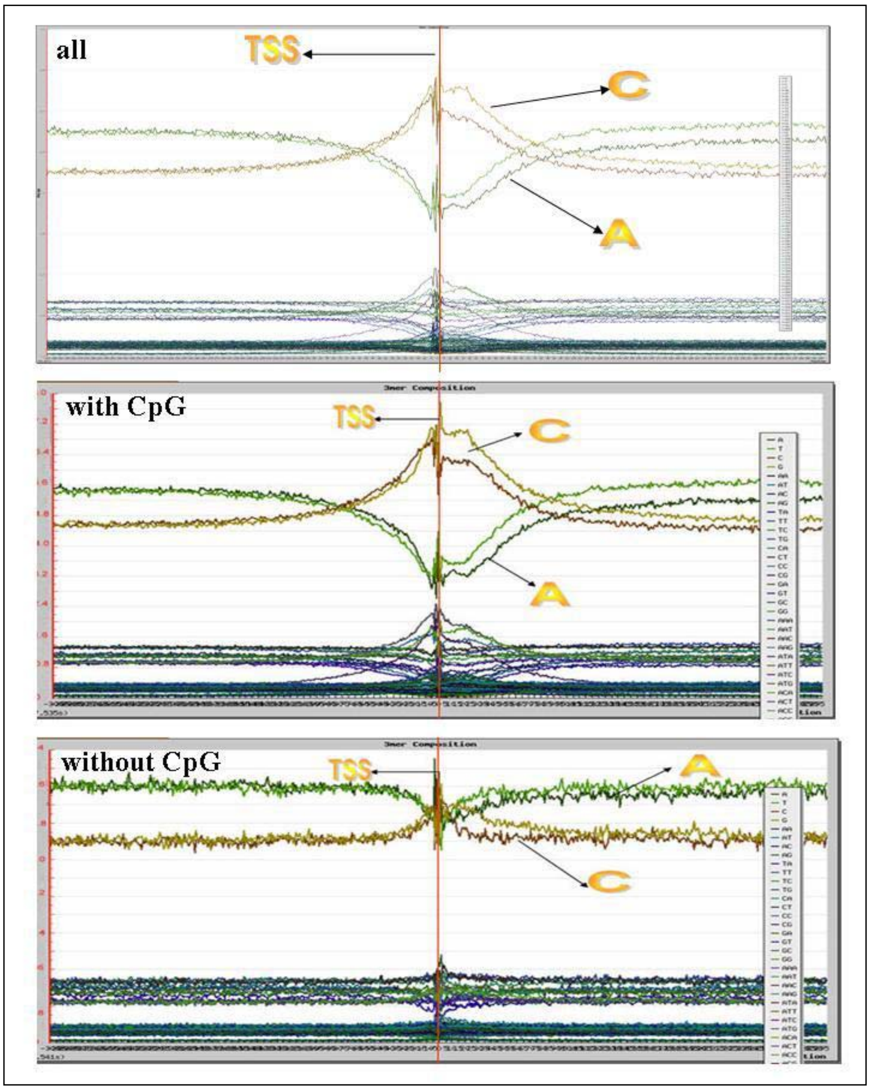

Figure S1. The average distributions of occurring rate of mono-, di-, and tri-mer nucleotides in human promoter training sequences (-3000 ~ +3000 of TSSs (+1)).

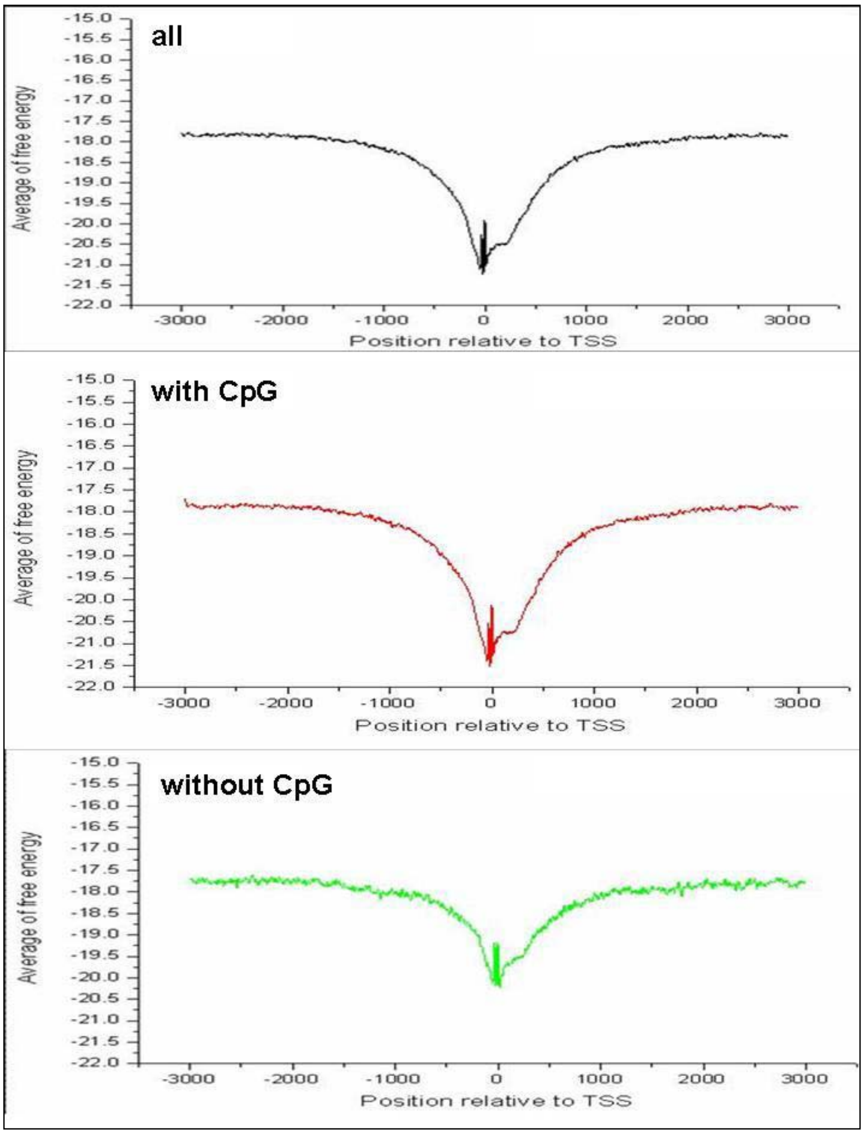

Figure S2. The average distributions of DNA stability in human promoter training sequences (-3000 ~ +3000 of TSSs (+1)). Near the TSS (+1), a peak lying between in the -10 to -30 bps region corresponds to the TATA box in the eukaryotic promoter sequences.

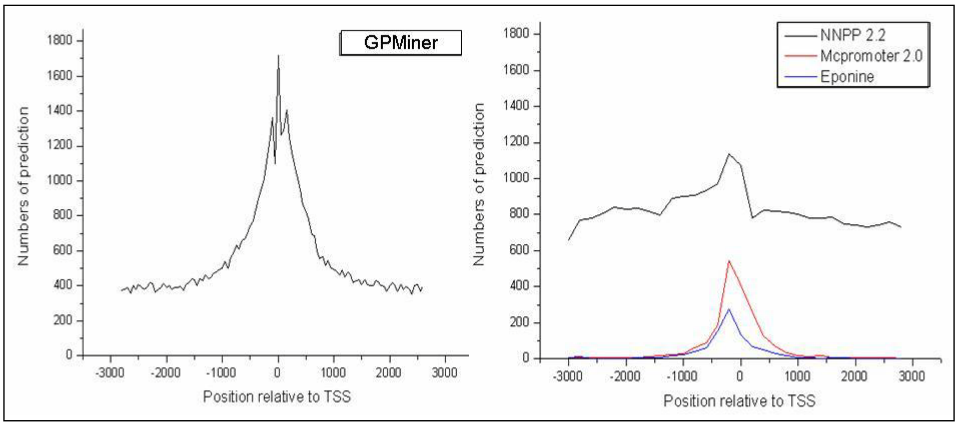

Figure S3. The distributions of promoter predictions of GPMiner comparing with NNPP2.2, Eponine, and McPromoter.

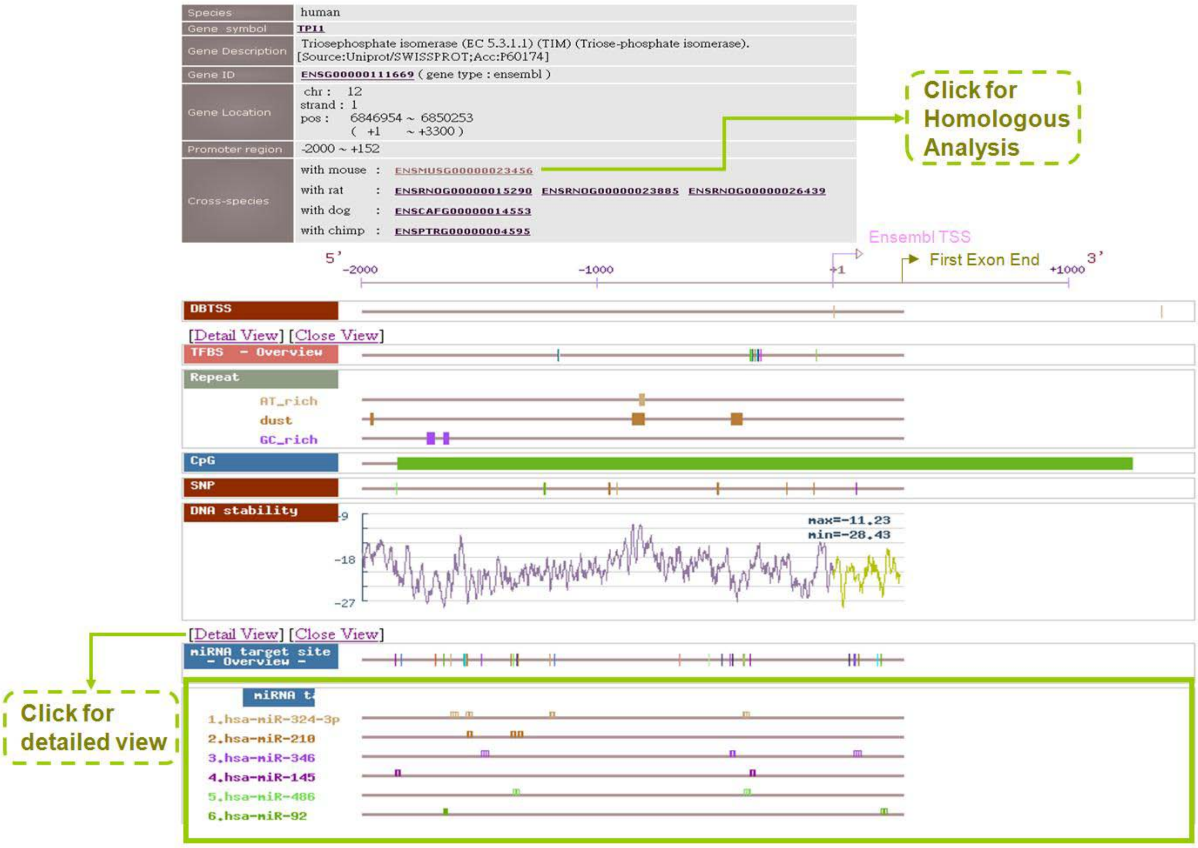

Figure S4. Graphical representation of regulatory elements for known gene promoter.

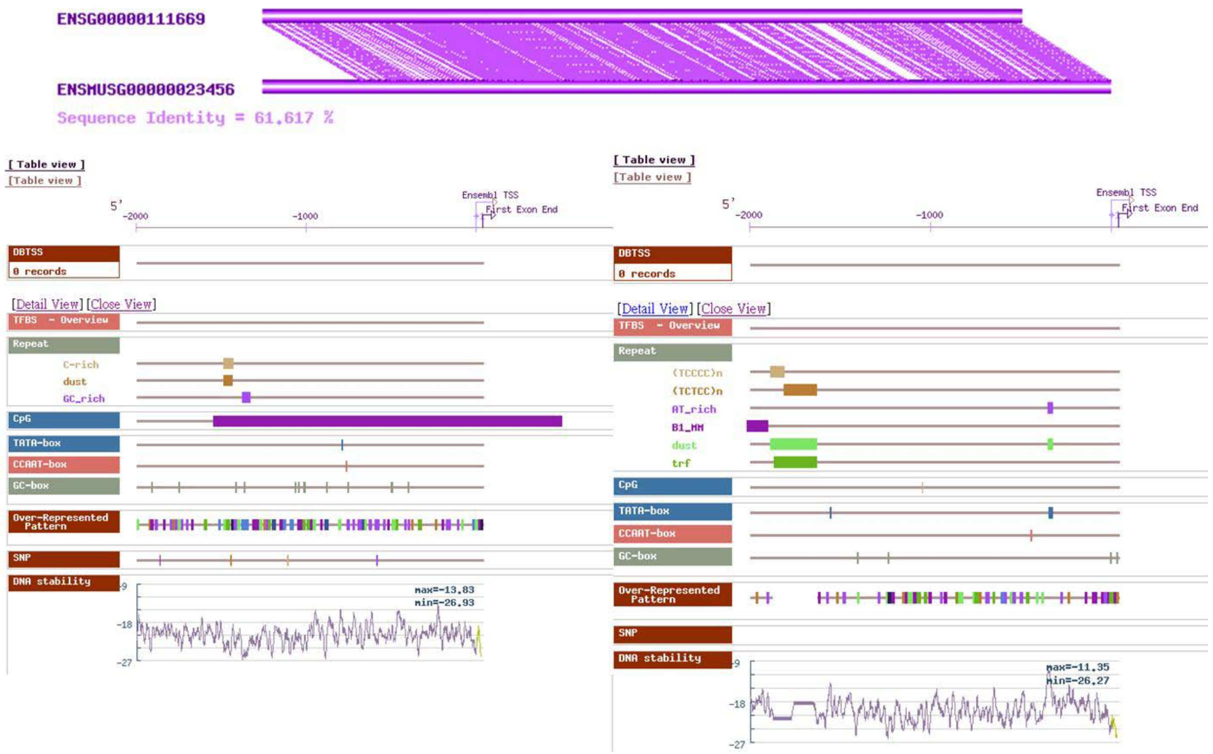

Figure S5. Graphical representation of regulatory elements for homologous promoter sequences.

REFERENCES

1. Kanhere, A. and M. Bansal, *A novel method for prokaryotic promoter prediction based on DNA stability.* BMC Bioinformatics, 2005. **6**(1): p. 1.
2. Kanhere, A. and M. Bansal, *Structural properties of promoters: similarities and differences between prokaryotes and eukaryotes.* Nucleic Acids Res, 2005. **33**(10): p. 3165-75.
3. Stabenau, A., et al., *The Ensembl core software libraries.* Genome Res, 2004. **14**(5): p. 929-33.
4. Thompson, J.D., D.G. Higgins, and T.J. Gibson, *CLUSTAL W: improving the sensitivity of progressive multiple sequence alignment through sequence weighting, position-specific gap penalties and weight matrix choice.* Nucleic Acids Res, 1994. **22**(22): p. 4673-80.
5. SantaLucia, J., Jr., *A unified view of polymer, dumbbell, and oligonucleotide DNA nearest-neighbor thermodynamics.* Proc Natl Acad Sci U S A, 1998. **95**(4): p. 1460-5.
